# Characterization and Differentiation of Spanish Vinegars from Jerez and Condado de Huelva Protected Designations of Origin

**DOI:** 10.3390/foods8080341

**Published:** 2019-08-12

**Authors:** Enrique Durán-Guerrero, Mónica Schwarz, M. Ángeles Fernández-Recamales, Carmelo G. Barroso, Remedios Castro

**Affiliations:** 1 Analytical Chemistry Department—IVAGRO, Faculty of Sciences, University of Cadiz, 11510 Puerto Real, Spain; 2 “Salus Infirmorum” Faculty of Nursing, University of Cadiz, 11001 Cadiz, Spain; 3 Department of Chemistry, Faculty of Experimental Sciences, University of Huelva, 21007 Huelva, Spain

**Keywords:** vinegar, volatile compounds, polyphenolic compounds, differentiation

## Abstract

Thirty one Jerez vinegar samples and 33 Huelva vinegar samples were analyzed for polyphenolic and volatile compound content in order to characterize them and attempt to differentiate them. Sixteen polyphenolic compounds were quantified by means of ultraperformance liquid chromatography method with diode array detection (UPLC–DAD), and 37 volatile compounds were studied by means of stir bar sorptive extraction–gas chromatography–mass spectrometry (SBSE–GC–MS). Spectrophotometric CIELab parameters were also measured for all the samples. The results obtained from the statistical multivariate treatment of the data evidenced a clear difference between vinegars from the two geographical indications with regard to their polyphenolic content, with Jerez vinegars exhibiting a greater phenolic content. Differentiation by the volatile compound content was, however, not so evident. Nevertheless, a considerable differentiation between the two groups of vinegars based on their volatile fraction was achieved. This may bring to light the grape varieties and geographical factors that have a clear influence on such differences.

## 1. Introduction

Vinegar has been used since ancient times as a condiment and as a food preservative, along with many other uses [1]. Spain, and more specifically Andalusia, has an important wine-making heritage. Different vinegars are made using specific methods, thanks to the particular climate and ambience conditions in the region as well as the traditional manufacturing techniques that have been kept unaltered for many years. Some of the vinegar varieties that are produced in this region using such traditional and specific methods are nowadays recognized as protected designation of origin (PDO) products, a geographical indication that guarantees to consumers their quality and origin as well as a control system that governs the products from their production at the vineyard up to their commercialization [2]. Vinegar from Jerez and Condado de Huelva are two examples of such PDO products.

Jerez vinegar must invariably be obtained from the acetic fermentation of wines produced at the wine-making geographical indication known as “Jerez-Xérès-Sherry” and “Manzanilla Sanlúcar de Barrameda”. The grape varieties that are allowed for vinegar-making are mainly Palomino and, to a lesser extent, Pedro Ximénez and Moscatel. These varieties are grown in the Jerez area and are adapted to its specific soil type, which is known as “albarizas”. They are also adapted to the rainfall conditions and to the prevailing wind and temperature ranges in the area. All of these characteristics make this geographical indication a unique region with regard to cultivating conditions [3].

Vinegars that have been granted Condado de Huelva PDO are produced exclusively from wines that have been awarded the same PDO and are produced and aged within that specific environment. Zalema is the main grape variety that has been authorized for the production of this PDO vinegar. The varieties Palomino Fino, Listán de Huelva, Garrido Fino, Moscatel de Alejandría, and Pedro Ximénez are also used to a lesser extent. Similar to Jerez PDO, wine and vinegar from Condado de Huelva are also special, thanks to the unique environmental conditions and the compulsory specific oenological practices employed in their production [4].

In order to study and improve wine vinegar quality, it is essential to focus on its bouquet, an already widely studied feature that characterizes its particular olfactory and flavor profile [5,6,7]. Apart from its obvious culinary contributions, its beneficial properties for a healthy diet are increasingly valued. Natural antioxidant compounds deserve a special mention as their antiviral and anti-inflammatory properties as well as their capacity to assist the regulation of blood pressure [8] have already been studied in Jerez vinegars [9,10,11].

Some studies have examined the volatile compounds in vinegars to distinguish and classify them according to their raw material, production processes, and origin using different analytical methods coupled to gas chromatography (GC), such as headspace (HS) [12], solid-phase extraction (SPE) [2], solid-phase microextraction (SPME) [13,14], stir bar sorptive extraction (SBSE) [15,16], and headspace sorptive extraction (HSSE) [17]. Vinegars have also been differentiated by their polyphenol content by means of liquid chromatography [15,18] or spectroscopic techniques, such as infrared (IR) [19,20,21] or fluorescence [22], just to mention some of them.

Jerez and Huelva are two relatively nearby production areas (both of them are in the southwest of Andalusia in Spain), and both use similar procedures and the same raw material (grapes) for the production of vinegar. Nevertheless, geographic and climate-specific conditions may still play a relevant role in the final product. In fact, there are some studies where Andalusian vinegars from different production areas have been differentiated by their volatile [17] or polyphenolic content [18]. The grape varieties that are employed for different Andalusian vinegars under a PDO may be varied, but it could be possible to find vinegars from the same grape variety that exhibit some differences that are attributable to the geographical area in which they have been produced. Úbeda et al. detected some differences in the volatile content of red wines even though all of them had been produced from Carignan grapes but from different areas within the Chilean region known as Maule [23].

This study intends to characterize and differentiate different vinegar varieties from two geographical indications—Jerez and Condado de Huelva—based on their volatile and polyphenolic content by means of stir bar sorptive extraction–gas chromatography–mass spectrometry (SBSE–GC–MS) and ultraperformance liquid chromatography method with diode array detection (UPLC–DAD), respectively. This could be a useful tool to ensure the characteristic profile and authenticity of the products that are protected by their respective geographical indications.

## 2. Materials and Methods

### 2.1. Vinegar Samples

A total of 64 vinegar samples (33 Condado de Huelva PDO vinegar samples and 31 Jerez PDO vinegar samples) were analyzed in duplicate. In both cases, the samples were supplied by a representative number of wine cellars from each PDO.

### 2.2. Spectrophotometric L*a*b* Measurements

Color measurements were made using a Helios UV–VIS spectrophotometer (Unicam, Cambridge, United Kingdom) over the visible range 380–770 nm, with each interval being 10 nm. Color analyses were carried out following the method of the International Commission on Illumination (CIE) [24]. The CIELab coordinates L*, a*, and b* were obtained using the D65 illuminant and a 10° observer. In this three-dimension system, the L* axis indicates the lightness whose value extends from 0 (black) to 100 (white), while a* and b* axes represent the chromaticity. Coordinate a* has positive values for red colors and negative values for green colors. Coordinate b* has positive values for yellow colors and negative values for blue colors.

### 2.3. Determination of Polyphenols and Furanic Compounds 

Polyphenol content was determined by UPLC according to the method improved by Schwarz et al. [25]. An Acquity UPLC equipment from Waters (Milford, MA, USA) coupled to a photodiode matrix ultraviolet–visible detector and an Acquity UPLC BEH C18 100 mm × 2.1 mm inner diameter column of 1.7 μm particle size (Waters, Milford, MA, USA). All the samples were filtered through 0.20 μm nylon filters (Millipore, Bedford, MA, USA) before their chromatographic analysis. In order to avoid possible losses of compounds during filtration, the amount of filtrated sample was large enough, and the initial volume was discharged.

The identification of each polyphenolic compound was carried out by comparing its retention time and ultraviolet–visible spectrum with those previously obtained from the analysis of standards. Calibration curves were established for each polyphenolic compound, with the concentration range taken into account according to bibliographic data.

The polyphenolic and furanic compounds that were quantified at 280 nm were as follows: gallic acid, 5-hydroximethyl-2-furaldehyde (HMF), furfural, tyrosol, syringic acid, protocatechuic acid, and protocatechuic aldehyde. The polyphenolic compounds quantified at 320 nm were as follows: caftaric acid, coutaric acid, caffeic acid, coumaric acid, syringaldehyde, ethyl caffeate, ethyl coumarate, and vanilline.

The polyphenolic compounds caftaric acid and ethyl caffeate were quantified by means of the caffeic acid calibration curve. Coutaric acid and ethyl coumarate were quantified by means of the coumaric acid calibration curve [25].

### 2.4. Determination of Volatile Compounds 

Volatile compounds were determined by means of SBSE–GC–MS according to the conditions proposed by Durán et al. [26]. An aliquot of sample (25 mL) was pipetted for each SBSE analysis and poured into a 100 mL Erlenmeyer flask containing 5.85 g of NaCl and 84 μL of a solution of 4-methyl-2 pentanol (2.27 g/L in Milli-Q water containing 80 g/L of acetic acid). The Erlenmeyer flask was placed in a 15-position magnetic agitator (Gerstel, Mülheim an der Ruhr, Germany). Magnetic stirring bars that were 10 mm long and 0.5 mm wide and covered by an absorbing polymer called polydimethylsiloxane (PDMS) were used for the extractions. Known in the market as Twisters^®^, they were supplied by Gerstel (Mülheim an der Ruhr, Germany). The stirring bars were agitated at 1250 rpm for 120 min at 25 °C. After extracting the bars from the vinegar samples, they were rinsed with distilled water for a few seconds to remove the salt remains, and they were then pat-dried using cellulose paper. Then, they were inserted in a glass tube to be thermally desorped.

After that, the stirring bars were thermally desorped by means of a TDS-2 thermal desorption unit (Gerstel, Mülheim an der Ruhr, Germany) connected by a heat transfer line to a vaporizing injector at a programmed temperature (PTV) known in the market as Cis-4 (Gerstel, Mülheim an der Ruhr, Germany). The PTV was installed onto an Agilent 6890 GC-5973N MS (Agilent, Little Falls, DE, USA) system fitted with a capillary column DBWax (J&W Scientific, Folsom, CA, USA) of 60 m × 0.25 mm inner diameter and a 0.25 µm thick coating.

The compounds were identified by means of the Wiley library according to their analogy with mass spectra and confirmed by their standard retention indices or by the retention data found in the literature. Semiquantitative data of the compounds that had been identified were obtained by measuring the relative area of each compound’s base peak (the most intense peak in the mass spectrum) in relation to the internal standard, 4-methyl-2-pentanol.

### 2.5. Statistical Analysis

The different data that were obtained were subjected to analysis of variance (ANOVA), principal component analysis (PCA), linear discriminant analysis (LDA), and cluster analysis (CA) by means of the computer application StatGraphics Centurion XVI (StatPoint Technologies, Inc., Warrenton, VA, USA).

## 3. Results and Discussion

### 3.1. Spectrophotometric L*a*b* measurements

L*a*b* parameters were measured for all the samples and ANOVA was performed. Table 1 shows the results obtained from the analysis.

As can be seen, only lightness (L*) showed significant difference for *p* < 0.05, with Sherry vinegars presenting higher values for this parameter. Although a* and b* parameters did not present significant differences, vinegars from Huelva showed higher values, so they were more close to green and blue colors than Sherry vinegars.

### 3.2. Furanic and Polyphenolic Compounds

Table 2 shows the average values (mg/L) determined for the different polyphenolic and furanic compounds found. A statistical analysis of the data collected was carried out in order to verify if the origin of the vinegar samples would have any relevant influence on their actual polyphenolic content. The origin of the vinegar samples was taken as the independent factor. According to the results obtained for the “origin” factor, shown in Table 2, there were significant differences between the two groups of vinegars studied when *p <* 0.01. In that respect, the compounds that met this requirement were as follows: gallic acid, tyrosol, syringic acid, furoic acid, caftaric acid, coutaric acid, caffeic acid, coumaric acid, syringaldehyde, ethyl caffeate, ethyl cumarate, and vanilline (Table 2). Similar values were obtained by other authors when Jerez and Huelva vinegars were analyzed [10,18]. It can be seen that several of these compounds could be found with a significantly higher content in Jerez vinegars.

The resulting data were subjected to principal component analysis. This statistical tool searches for new variables to explain the maximum measured variability (polyphenols) between the different samples at the same time by taking into account the different correlations between them. The PCA is a statistical method that allows the number of variables to be reduced with the smallest possible amount of information getting lost in the process. The new principal components or factors are then expressed as a linear combination of the original variables and are also independent from each other. This allows the total variability to be explained while making use of a reduced number of factors.

**Figure 1:**
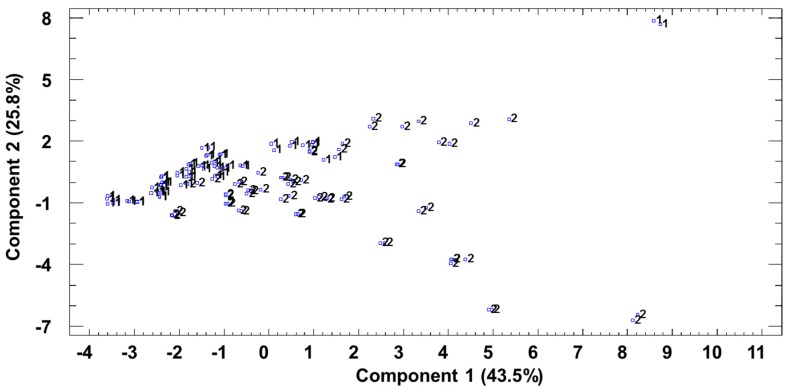
Principal component analysis of polyphenolic and furanic compounds. Projection of the samples onto the plane formed by the first two principal components. 1—Huelva; 2—Jerez.

According to Kaiser’s criterion, three principal components were extracted to explain 78% of the variance between the samples. When Kaiser’s criterion is applied, only components with eigenvalues greater than 1.00 are retained and interpreted. This is one of the most commonly used criterion and was proposed by Kaiser in 1960 [27]. The graphical representation of the samples on the orthogonal plane defined by the first two principal components can be seen in Figure 1. It can also be seen that PC1 was different depending on the geographical indication and that this PC took a positive value in the case of Jerez vinegars. The compounds with the greatest influence on PC1 were tyrosol, syringic acid, *p*-coumaric acid, syringaldehyde, and ethyl caffeate. The content of these polyphenols clearly increases when the vinegars are kept in wood containers, and this is something even more noticeable in Jerez vinegars [14].

A forward stepwise linear discriminant analysis with the leave-one-out cross-validation method was carried out later on. This type of analysis establishes which of the measured variables—in this case, polyphenolic compounds—contributes by a greater extent to a successful discrimination according to the geographical indication of each vinegar sample. Almost all the Huelva vinegar samples (96.88%) and 88.71% of the Jerez ones were successfully discriminated, meaning 92.86% of all samples were successfully classified. The compounds with the highest influence on the discriminant function were caftaric acid, *p*-coutaric acid, furfural, and syringaldehyde, among others.

**Figure 2:**
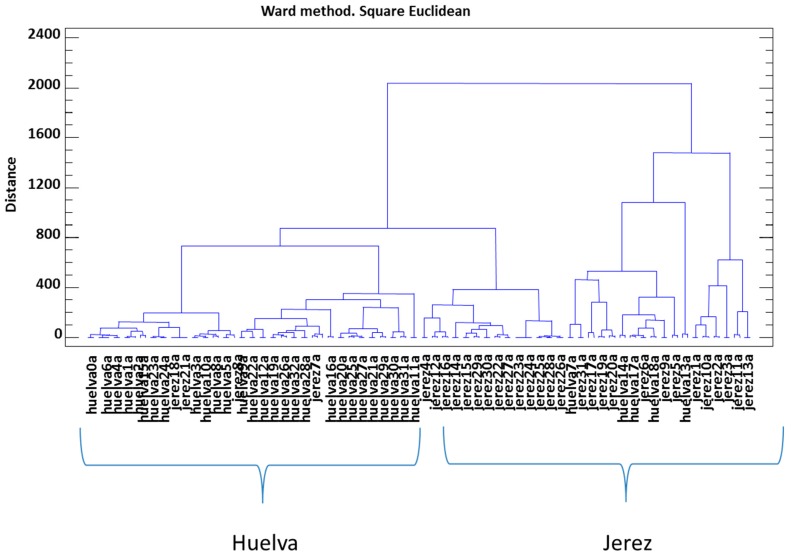
Cluster analysis of polyphenolic and furanic compounds.

Finally, the data on polyphenolic compounds were subjected to cluster analysis using Ward’s method for clustering and the Euclidean square distance metric for comparison. This is a statistical multivariate technique that clusters elements (or variables) to obtain the greatest possible homogeneity within each group on the one hand and the greatest possible difference between groups on the other. The result is represented by a classification tree or dendogram in Figure 2.

It can be seen that two clear clusters were obtained, one with a large number of samples of Jerez vinegars and the other with Huelva vinegars. Nevertheless, some of the Jerez vinegar samples were classified together with Huelva vinegars. This fact may highlight how similar both groups are between them.

### 3.3. Volatile Compounds

Similar to the previous study on polyphenolic compounds, the analysis of variance based on a significant factor, i.e., the origin of the vinegar samples, was also carried out for the geographical indications Huelva and Jerez. Table 3 shows the results obtained from the ANOVA.

It can be observed that only a few of the volatile compounds demonstrated relevant differences in a single variance approach between the two geographical indications when the significance level was *p* < 0.01. These were 4-ethylguaiacol, 4-ethylphenol, 5-acetoxymethyl-2-furaldehyde, octanoic acid, decanoic acid, isobutanol (*p* < 0.05), isopentyl acetate, 3-hydroxy-2-butanone (*p* < 0.05), isovaleric acid, hexanoic acid (*p* < 0.05), and 2-phenylethanol. They exhibited a significant difference (*p* < 0.01) between vinegars from each of the two geographical indications, with generally higher value for vinegars from Jerez protected geographical indication (Table 3).

Subsequently, a PCA was carried out in order to cluster the differences in volatile content within a smaller number of variables (PC), which would explain the maximum variability between the samples. According to Kaiser’s criterion (eigenvalue >1), 11 principal components were extracted, which explained 82% of the variance between the samples. The first two PCs accounted for 42.14% of the variability.

**Figure 3:**
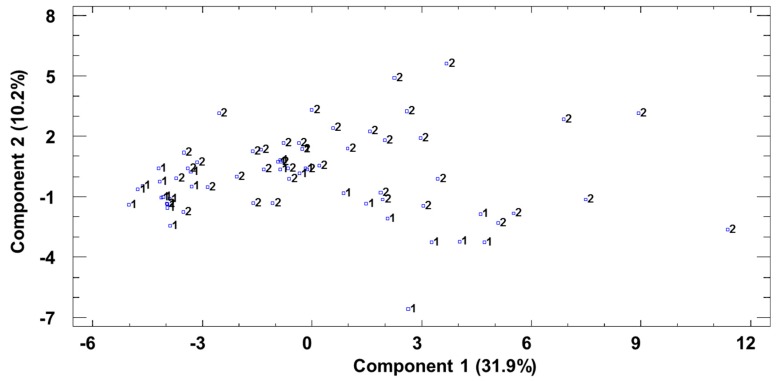
Principal component analysis of volatile compounds. Projection of the samples onto the plane formed by the first two principal components. 1—Huelva; 2—Jerez.

The orthogonal representation of the samples was used to observe the distribution of the data with regard to PC1 and PC2 (Figure 3). The distribution of the samples on this graphical representation did not show any clear differences between the vinegars from either origin, although Huelva vinegar samples did appear on the lower area of PC2.

The volatile compounds with a greater influence on PC1 were as follows: *n*-butyl acetate, 2-acetyl-5- methylfurfural, 4-ethylguaiacol, 4-ethylphenol, octanoic acid, decanoic acid, isopentyl acetate, 2-methyl-1-butanol, 3-methyl-1-butanol, ethyl octanoate, ethylphenyl acetate, and phenylethanol.

With regard to PC2, the compounds that demonstrated a greater difference between the samples were as follows: 4-ethylguaiacol, 4-ethylphenol, ethyl-2-methylbutyrate, hexanal, isovaleric acid, and hexanoic acid.

A backward stepwise linear discriminant analysis was carried out later on. In this case, the percentage of correctly classified samples was 100% for Huelva vinegars and 90.24% for Jerez vinegars. After calculating the discriminant function, only three of the samples were misclassified, with only one of the samples being made from Pedro Ximénez grapes. The percentage of samples correctly classified reached up to 94.12% altogether. The compounds that displayed coefficients with the greatest influence according to the discriminating function were 3-methyl-1-butanol, benzaldehyde, ethyl hexanoate, hexyl acetate, and 4-ethylguaiacol, among others. Other authors have successfully differentiated vinegar types, including Jerez and Huelva vinegars, according to their volatile content [17]. However, the volatile compounds with a greater influence on the discrimination in such studies were different from the ones obtained in the present study because these authors studied different volatile compounds.

**Figure 4:**
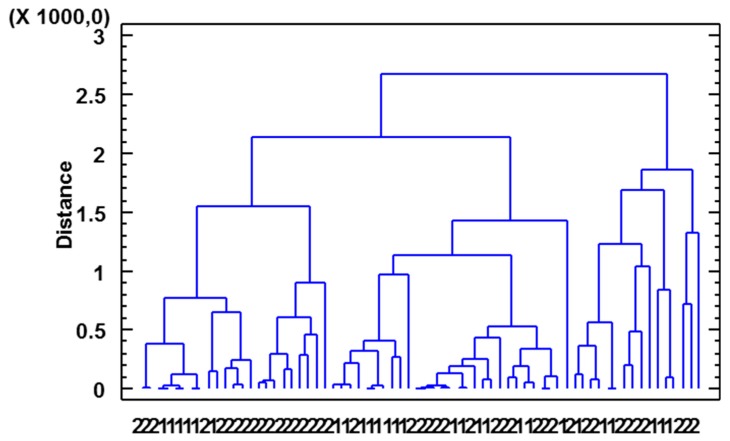
Cluster analysis of volatile compounds. 1—Huelva; 2—Jerez.

When the data on volatile compounds were subjected to cluster analysis using Ward’s method and the Euclidean square distance metric as comparison criterion, the results failed to establish a clear difference between the two geographical indication, i.e., vinegars from both regions were arranged within the same groups, as can be seen in Figure 4.

### 3.4. Joint Analysis of Polyphenolic, Furanic, and Volatile Constituents

Finally, in order to determine the most relevant variables for the differentiation of vinegars from the two PDOs, a principal component analysis was carried out in which the polyphenolic, furanic, and volatile constituents were taken into account.

**Figure 5:**
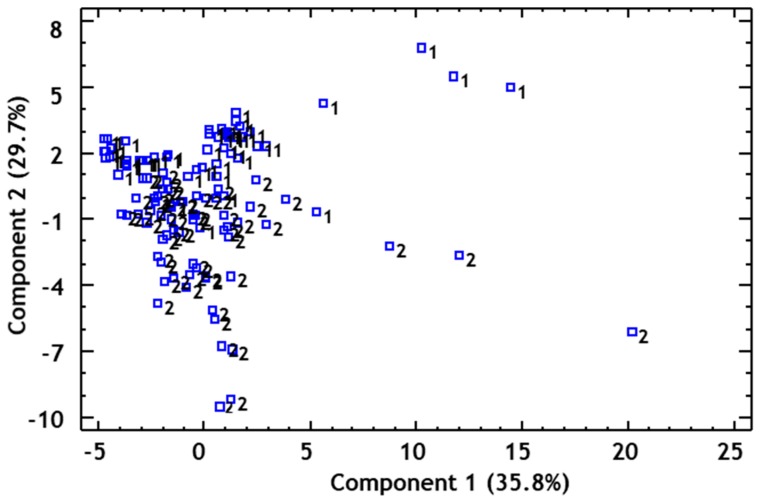
Principal component analysis of volatile, polyphenolic, and furanic compounds. Projection of the samples onto the plane formed by the first two principal components. 1—Huelva; 2—Jerez.

In this case, 11 PCs (eigenvalue >1) were obtained to justify 88.8% of the samples’ inherent variability. The first two PCs accounted for 53.5% of the variability. When the different samples were represented on the plane defined by these first two principal components (Figure 5), it could be observed that PC2 was the one that clearly differentiated Jerez vinegar (2) from Huelva ones (1), with negative values of this PC for Jerez vinegars.

In this case, the polyphenolic and furanic compounds showed a clearly greater contribution to this principal component, with the hydroxycinnamic derivates (caftaric acid, *p*-coutaric acid, caffeic acid, *p*-coumaric acid, ethyl caffeate, and ethyl coumarate) as the variables with the greatest weight; all of them had a negative sign. With regard to the volatile compounds that contributed to this PC, 4-ethylguaiacol, propyl acetate, ethyl isobutyrate, ethyl isovalerate, 1-hexanol, and benzyl alcohol are worth mentioning.

## 4. Conclusions

The statistical study of polyphenols and volatile compounds in Jerez and Huelva PDO vinegars demonstrated clear differences between these two geographical indications. The differences were clearly based on their polyphenolic compound content. As vinegar manufacturing and ageing processes are similar in both regions, we should presume the importance of other factors, such as the grape varieties used as well as other geographical factors.

## Figures and Tables

**Table 1:** CIELab. Mean values and standard deviations of the parameters and analysis of variance.

	Jerez	Huelva	*F*-Ratio	*p*-Value
L* ^+^	92.09 ± 3.24	89.44 ± 6.57	4.09	0.0476
a*	0.82 ± 1.17	1.73 ± 2.45	3.49	0.0664
b*	14.56 ± 7.54	19.46 ± 13.83	3.03	0.0866

^+^ Significant differences for *p* < 0.05.

**Table 2:** Polyphenolic and furanic compounds. Mean values of concentrations (mg/L) and analysis of variance.

Polyphenolic Compounds	Jerez (mg/L)	Huelva (mg/L)	*F*-Ratio	*p*-Value
Gallic acid *	50.7	36.7	7.78	0.0061
5-Hydroxymethyl-2-furaldehyde	70.9	66.9	0.04	0.84
Furfural	56	77.9	6.62	0.0112
Tyrosol *	20.8	15.6	14.25	0.0002
Syringic acid *	11.7	6.5	25.96	0
Protocatechuic acid	58.2	67.8	1.94	0.1661
Furoic acid *	64.5	172.6	34.36	0
Caftaric acid *	14.3	3.2	42.34	0
Coutaric acid *	13.5	3.4	73.59	0
Caffeic acid *	6.6	2.2	43.83	0
p-Coumaric acid *	8.1	1.7	71.36	0
Syringaldehyde *	7.3	3.6	50.84	0
Protocatechuic aldehyde	1.8	1.6	0.51	0.4785
Ethyl caffeate *	1.5	0.5	20.62	0
Ethyl coumarate *	1.2	0.1	20.62	0
Vanilline *	4.3	3	13.34	0.0004

* Significant differences for *p* < 0.01.

**Table 3:** Volatile compounds. Mean values of relative areas (RA) and analysis of variance.

Volatile Compounds	Jerez (RA)	Huelva (RA)	*F*-Ratio	*p*-Value
Eugenol	0.0121	0.0135	0.52	0.4735
Ethyl pentanoate	0.049	0.0047	67	0.4144
Ethyl lactate	1.9712	3.8071	0	0.9513
n-Butyl acetate	2.1283	1.1981	3.76	0.0566
*trans*-2-Hexen-1-ol	0.0074	0.0318	2.43	0.124
Furfural	0.0283	0.0282	2.15	0.147
Isobutyric acid	0.1154	0.4554	0.16	0.6948
2-Acetyl-5-methylfurfural	0.0171	0.013	0.02	0.8935
4-Ethylguaiacol *	0.1262	0.0141	48.24	0
4-Ethylphenol *	0.2041	0.0279	31.79	0
5-Acetoxymethyl-2-furaldehyde *	0.0311	0.0056	8.67	0.0044
Octanoic acid *	1.1339	0.6532	21.45	0
Decanoic acid *	1.7839	1.1319	12.4	0.0008
Ethyl isobutyrate	0.3935	0.4827	0.12	0.7349
Propyl acetate	0.0389	1.8364	1.8	0.1837
Isobutyl acetate	2.2364	4.7232	1.99	0.1626
Ethyl 2-methylbutyrate	0.1942	0.2033	0.87	0.3545
Ethyl isovalerate	1.853	1.7505	2.37	0.1281
Hexanal	0.0293	0.1588	0.64	0.4247
Isobutanol **	0.0155	0.5006	5.67	0.02
Isopentyl acetate *	7.8289	3.309	9.34	0.0032
2-Methyl-1-butanol	0.402	0.2769	1.07	0.3052
3-Methyl-1-butanol	0.5129	0.3514	0.45	0.5066
Ethyl hexanoate	0.2035	0.1724	0.24	0.6227
Hexyl acetate	0.0271	0.0262	0.03	0.8537
3-Hydroxy-2-butanone **	0.0279	0.0486	5.85	0.0183
*cis*-3-Hexenylacetate	0.0318	0.1708	3.51	0.0653
Hexanol	0.01	0.011	0.67	0.4175
Ethyl octanoate	0.2668	0.1358	0.89	0.3497
Benzaldehyde	0.1167	0.065	0	0.9476
Isovaleric acid *	1.1198	0.4681	11.69	0.0011
Diethyl succinate	0.1328	0.0899	0.52	0.4713
α-Terpineol	0.0025	0.0015	0.31	0.3341
Benzyl acetate	0.1321	0.0426	2.65	0.1081
Ethylphenyl acetate	1.4938	1.0218	3.77	0.0563
Hexanoic acid **	0.3401	0.1818	6.67	0.012
Phenylethanol *	1.5502	0.5302	8.91	0.0039

* Significant differences for *p* < 0.01. ** Significant differences for *p* < 0.05.
